# Nitric Oxide-Driven Hypoxia Initiates Synovial Angiogenesis, Hyperplasia and Inflammatory Lesions in Mice

**DOI:** 10.1371/journal.pone.0034494

**Published:** 2012-03-30

**Authors:** Fei Bao, Pei Wu, Na Xiao, Frank Qiu, Qing-Ping Zeng

**Affiliations:** 1 Tropical Medicine Institute, Guangzhou University of Chinese Medicine, Guangzhou, China; 2 Simplex Biotechnologies, LLC, Clinton, New Jersey, United States of America; Institut Jacques Monod, France

## Abstract

**Background:**

Rheumatoid arthritis (RA) is an inflammatory articular disease with cartilage and bone damage due to hyperplasic synoviocyte invasion and subsequent matrix protease digestion. Although monoclonal antibodies against tumor necrosis factor alpha (TNFα) have been approved for clinical use in patients with RA, desired therapeutic regimens suitable for non-responders are still unavailable because etiological initiators leading to RA remain enigmatic and unidentified.

**Methodology/Principal Findings:**

Bacteria-induced arthritis (BIA) that simulates collagen-induced arthritis (CIA) is developed in mice upon daily live bacterial feeding. The morphological lesions of paw erythema and edema together with the histological alterations of synovial hyperplasia and lymphocytic infiltration emerge as the early-phase manifestations of BIA and CIA. Bacteria- or collagen-mediated global upregulation of pro-inflammatory cytokines is accompanied by the burst of nitric oxide (NO). Elevation of the serum NO level is correlated with decline of the blood oxygen saturation percentage (SpO2), reflecting a hypoxic consequence during development towards arthritis. NO-driven hypoxia is further evident from a positive relationship between NO and lactic acid (LA), an end product from glycolysis. Upregulation of hypoxia inducible factor 1 alpha (HIF-1α) and vascular endothelial growth factor (VEGF) validates hypoxia-induced angiogenesis in the inflamed synovium of modeling mice. Administration of the NO donor compound sodium nitroprusside (SNP) causes articular inflammation by inducing synovial hypoxia. Anti-bacteria by the antibiotic cefotaxime and/or the immunosuppressant rapamycin or artesunate that also inhibits nitric oxide synthase (NOS) can abrogate NO production, mitigate hypoxia, and considerably ameliorate or even completely abort synovitis, hence highlighting that NO may serve as an initiator of inflammatory arthritis.

**Conclusions/Significance:**

Like collagen, bacteria also enable synovial lesions via upregulating pro-inflammatory cytokines, triggering NO production, driving hypoxic responses, and inducing synovial angiogenesis and hyperplasia, suggesting that sustained infection might be, in part, responsible for the onset of synovitis and arthritis in mice.

## Introduction

Rheumatoid arthritis (RA) is a chronic articular inflammatory disease mainly affecting joints and destroying cartilage and bone, often with severe and disabling consequences [1]. RA also affects lungs, pleura, pericardium, sclera and subcutaneous tissue [2], so patients with RA have an elevated risk in developing cardiovascular diseases, such as arteriosclerosis and myocardial infarction [3]. Histopathologically, RA is characterized by pronounced synovial hyperplasia, or called pannus, a thickened membrane-like covering of the inflammatory granulation tissue over the articular cartilage. Like a malignant tumor, the pannus can invade and destroy cartilage and bone by secreting matrix proteases such as metalloproteinases and aggrecanases [4].

Although monoclonal antibody-based biologic agents that inhibit tumor necrosis factor alpha (TNFα), including etanercept, infliximab, adalimumab, golimumab, and certolizumab, have been licensed for clinical use in patients with RA [5], [6], approximately 40% of RA patients that have accepted those anti-TNFα antibodies are non-responders. Importantly, inactivation of TNFα interferes with innate immune defense and predisposes a risk of pathogenic infection. Moreover, joint repair and erosion healing are rare despite effective therapies with TNFα inhibitors [7], [8]. Until now, therapeutic regimens sensitive, effective and suitable for non-responders are unavailable because no etiological initiators leading to RA have been validated. Given these facts that TNFα is produced upon exposure to bacterial components such as lipopolysaccharide (LPS) and other endotoxins, TNFα is stimulated by microbial pathogens for orchestrating anti-microbial responses, and TNFα inhibitory biologic agents render users at a raised risk of serious infection [9]–[11], it is conceivable that TNFα blockers or antagonists should ameliorate RA by abolishing infection-evoked TNFα, and also logically reasonable that the onset of RA is likely attributed, in part, to microbial pathogens.

Microorganisms have been implicated as the cause of many rheumatic diseases, but there is no evidence supporting that infectious agents are directly involved [12]. Most recently, a surprising finding has emerged that the commensal Gram-positive segmented filamentous bacteria (SFB) drives an autoimmune disease in K/BxN mice with disease being abrogated under germ-free conditions and restored after colonization with SFB [13]. To this finding, a commentary annotation has been given that gut microbiota-induced overproduction of interleukins (IL-1, IL-6, IL-17, IL-22, and IL-23) may spill into systemic circulation and promote autoimmune attacks at distant sites, such as joints [14]. Therefore, gut infection-activated interleukines are directly linked to autoimmune-related articular lesions. We argue that, however, interleukines are unlikely relevant to synovial hyperplasia seen in RA, implying that alternative inducer(s) may exist to accelerate tumor-like proliferation in the synovium.

A central role of nitric oxide (NO) in the pathogenesis of RA has been previously suggested and currently pinpointed, but the revealed mechanism is only restricted in NO-mediated immune dysfunction [15], [16]. From clinical data, we know that the inflamed synovium is a predominant source of NO in patients with RA, and T cells from RA patients produce 2.5 times more NO than the healthy donor T cells [17], [18]. Experimentally, blockade of TNFα downregulates NO synthase (NOS) in human peripheral blood mononuclear cells [19]. An engineered peptide of the growth factor progranulin (PGRN), Atsttrin, is therapeutic against inflammatory arthritis in mice through binding to TNF receptors to inhibit TNFα-dependent NO production from macrophages [20]. Triptolide extracted from *Tripterygium wilfordii* Hook F is effective for treatment of experimental arthritis, probably due to inhibition of NOS by this compound [21]. From all above results and other references regarding bacterial infection-induced NOS in human neutrophils [22] and NO-driven angiogenesis and carcinogenesis [23], [24], we assume that NO may be a candidate mediator initiating synovial hyperplasia. Until recently, however, cumulative evendence concerning NO-induced tumor-like synovial hyperplasia is trivial, and the *bona fide* mechanism behind how NO drives synovial hyperplasia also remains completely undefined.

To figure out a possible association of gastrointestinal bacterial infection with inflammatory arthritis, we established a mouse model of bacteria-induced arthritis (BIA) that simulates collagen II (CII)-complete Freund's adjuvant (CFA)-induced arthritis (CIA) by daily live bacterial feeding. Dynamic changes of the serum level of NO, along with the blood saturation percentage of oxygen (SpO2) and the serum level of lactic acid (LA) were compared among modeling mice. Furthermore, quantitative profiling of 40 kinds of pro-inflammatory cytokines and immunoquantification of angiogenesis-relevant hypoxia inducible factor 1 alpha (HIF-1α) and vascular endothelial growth factor (VEGF) were performed during modeling. Importantly, a conclusion indicating the potential implication of NO in articular inflammation had been drawn based on the result of an acute synovitis induced by the NO donor compound sodium nitroprusside (SNP). Finally, we investigated whether synovial inflammation could be compromised when infection- or immunization-triggered NO was abolished by administration of the antibiotic cefotaxime and/or the immunosuppressant rapamycin or artesunate. The present study pays attention to elucidate whether sustained gastrointestinal bacterial infection would represent one of the pathogenic initiators towards RA, and to answer how NO could serve as a pivotal signal convey bacterial infection to articular inflammation.

## Results

### Inflammatory articular lesions were correlated with synovial hyperplasia and lymphocytic infiltration

**Figure 1:**
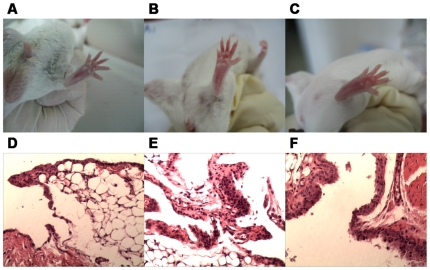
Morphological inflammation of erythematous and edematous paws and histochemical lesions of synovial hyperplasia and inflammatory infiltration in BIA and CIA mice. A and D. Control; B and E. BIA mice; C and F. CIA mice. HE staining of sections and microscopic analysis were carried out by sampling hind paws of mice after modeling for 28 d. The histochemical photographs were amplified for 100 folds.

The early-phase inflammatory articular lesions, mainly paws becoming red and swollen, emerged after four-week daily feeding of mice with live bacteria (about 10^8^) derived from overnight cultures of the non-pathogenic *E. coli* strain DH5α. In similar, intra-dermal injection of mice with CII-CFA by primary challenging on the 1st d and boosting on the 21st d also led to redness and swelling of paws within four weeks (Figure 1A, B, and C). CIA mice seemed to have developed more significant inflammatory arthritis than BIA mice, suggesting that CII-CFA was more effective than bacteria for modeling of experimental arthritis in mice. While the severity was scored as eight in total or two for each in CIA mice, only half of severity scores, i.e., four in total or one for each, could be recorded for BIA mice. Nevertheless, this result indicated that prolonged live bacterial feeding could mimick CII-CFA immunization to cause a typical, albeit mild, arthritic phenotype in mice.

To further identify the pathological alterations occurring in the articulate of modeling mice, we conducted histopathological analysis of the synovial tissue of BIA and CIA mice. In histochemical sections of the inflamed synovium, no articular damage and subintimal fibrosis were found, but multi-layer intimal hyperplasia and mild synovial infiltration were observed (Figure 1D, E, and F). Although the inflammatory extent in terms of lymphocytic infiltration was nearly identical in both BIA and CIA mice, a higher degree of intimal hyperplasia was exhibited by CIA mice than BIA mice (Table 1). The histological alteration of synovial hyperplasia was, therefore, mirrored in the phenotypical manifestation of inflammatory events. This result revealed again that persistent gut infection, even by non-pathogenic bacteria, also provoked the onset of synovitis in susceptible mice.

**Table 1:** Semi-quantitative evaluation of histological damage by a modified non-parametric scoring system in BIA and CIA mice.

	Intimal hyperplasia	Subintimal fibrosis	Lymphocytic infiltration	Articular damage
Control	0	0	0	0
BIA	+++	0	+++	0
CIA	++++	0	+++	0

Note: The histological damage was scored after 28 d for articular sections of modeling mice, in which BIA was induced by daily bacterial feeding; and CIA was developed by twice immunizations (primary challenge and boosting).

**Figure 2:**
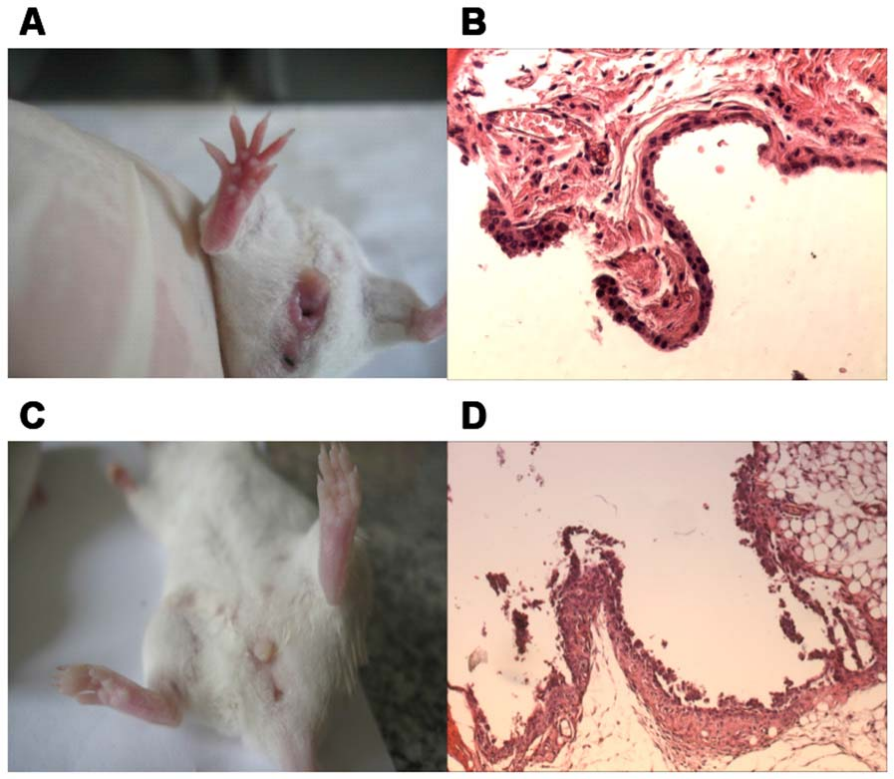
Morphological inflammation of erythematous and edematous paws and histochemical lesions of synovial hyperplasia and inflammatory infiltration in BIA-CIA mice. A and B. BIA-CIA mice (intra-dermal CII-CFA injection); C and D. BIA-CIA mice (intra-articular CII-CFA injection). HE staining of sections and microscopic analysis were carried out by sampling hind paws of mice after modeling of BIA-CIA mice (intra-dermal CII-CFA injection) for 28 d and after modeling of BIA-CIA mice (intra-articular CII-CFA injection) for 3 d, respectively. The histochemical photographs were amplified for 100 folds.

Surprisingly, BIA-CIA mice established by live bacterial feeding and synchronous intra-dermal CII-CFA injection was eventually developed into synovitis after four weeks, but with only mild morphological lesions and less histological damage (Figure 2A and B). While CIA mice showed more severe lymphocytic infiltration, BIA-CIA mice exibited only mild lymphocytic infiltration. Additionally, co-treatment of mice by intra-articular CII-CFA injection with live bacterial feeding also led to alleviated articular inflammation and histological alterations within 3 d (Figure 2C and D). These results implied that bacteria might exert an immunosuppressive effect on CII-CFA-elicited immune activation in BIA-CIA mice.

### Cytokine antibody profiling validated global upregulation of pro-inflammatory cytokines upon infection or immunization

**Figure 3:**
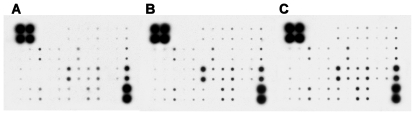
Antibody chip profiling of pro-inflammatory cytokines in blood of BIA, CIA and BIA-CIA mice. A. BIA mice; B. CIA mice; C. BIA-CIA mice. For protein extraction and antibody hybridization, whole blood was collected from mice after modeling for 28 d, and 40 kinds of cytokines, chemokines and receptors were quantitatively analyzed.

To follow up the immunological profile during the pathogenesis of arthritis, we conducted antibody chip analysis on as many as 40 kinds of common cytokines, chemokines and receptors in BIA, CIA and BIA-CIA mice (Figure 3A, B, and C). As results, all tested pro-inflammatory cytokines were almost upregulated with some downregulated in BIA and CIA mice, in which the most important pro-inflammatory cytokines including interferon γ (IFNγ), interleukins (IL), and colony-stimulating factors (CSF) were upregulated. However, BIA-CIA mice showed an overall downregulation of such pro-inflammatory cytokines as IFNγ, TNFα, IL-1β, IL-6, and IL-10 (Table 2). These results revealed a direct association of pro-inflammatory cytokines with inflammatory arthritis.

**Table 2:** Global modulation of inflammation-related cytokines in blood of BIA, CIA and BIA-CIA mice.

Name of cytokines	Folds of BIA to Control	Up-/down-regulation	Folds of CIA to Control	Up-/down-regulation	Folds of BIA-CIA to Control	Up-/down-regulation
BLC	1.86	↑	1.52	↑	1.28	↑
CD30 L	1.88	↑	1.63	↑	0.90	↓
Eotaxin	2.11	↑	1.97	↑	0.85	↓
Eotaxin-2	1.51	↑	1.56	↑	1.02	↑
Fas Ligand	1.69	↑	1.53	↑	0.58	↓
Fractalkine	1.85	↑	1.69	↑	0.98	↓
G-CSF	1.66	↑	1.46	↑	1.21	↑
GM-CSF	1.54	↑	1.43	↑	1.22	↑
IFNγ	1.12	↑	1.27	↑	0.93	↓
IL-1α	1.00	↓	0.95	↓	0.75	↓
IL-1 β	0.71	↓	1.14	↑	0.47	↓
IL-2	1.05	↑	1.08	↑	0.55	↓
IL-3	0.80	↓	0.95	↓	0.49	↓
IL-4	1.62	↑	1.31	↑	0.98	↓
IL-6	1.58	↑	1.22	↑	0.08	↓
IL-9	1.73	↑	1.60	↑	0.68	↓
IL-10	1.88	↑	1.20	↑	0.36	↓
IL-12p40p70	2.05	↑	1.89	↑	0.87	↓
IL-12p70	1.23	↑	1.06	↑	0.90	↓
IL-13	1.49	↑	0.94	↓	1.39	↑
IL-17	1.07	↑	1.67	↑	0.23	↓
I-TAC	2.02	↑	2.22	↑	1.27	↑
KC	1.86	↑	1.75	↑	0.74	↓
Leptin	1.73	↑	1.35	↑	1.01	↑
LIX	1.19	↑	1.27	↑	1.22	↑
Lymphotactin	1.93	↑	1.38	↑	0.98	↓
MCP-1	1.75	↑	1.53	↑	0.95	↓
M-CSF	1.41	↑	1.23	↑	0.88	↓
MIG	2.04	↑	1.37	↑	0.81	↓
MIP-1α	1.64	↑	1.31	↑	0.98	↓
MIP-1γ	0.99	↓	0.96	↓	1.04	↑
RANTES	2.03	↑	1.40	↑	1.88	↑
SDF-1	1.44	↑	1.29	↑	0.91	↓
TCA-3	1.50	↑	1.18	↑	0.88	↓
TECK	2.60	↑	1.53	↑	0.55	↓
TIMP-1	2.40	↑	1.42	↑	0.79	↓
TIMP-2	1.66	↑	0.89	↓	0.66	↓
TNFα	2.96	↑	0.59	↓	0.25	↓
sTNF RI	1.88	↑	1.42	↑	1.27	↑
sTNF RII	1.87	↑	1.53	↑	2.23	↑

Note: The antibody-based microarry analysis of cytokines, chemokines, and receptors was conducted after 28 d for blood of modeling mice, in which BIA was induced by daily bacterial feeding; CIA was developed by twice immunizations (primary challenge and boosting); and BIA-CIA was established by daily bacterial feeding and twice immunizations (primary challenge and boosting).

TNFα represents a critical pro-inflammatory cytokine, but it was not simultaneously upregulated in both BIA and CIA mice. As seen from Table 2, TNFα was upregulated for 2.96 folds in BIA mice, but downregulated to 60% in CIA mice. It was suggested that the downregulation of TNFα in CIA mice might be resulted from the decay of immune responses to earlier immunization because several days had passed from CII-CFA injection to cytokine profiling. On the other hand, TNFα receptors, sTNF RI and sTNF RII, were upregulated in both BIA and CIA mice, seeming to imply that TNF-TNFR signaling was actually enhanced. In similar, sTNF RI and sTNF RII were among upregulated cytokine receptors in BIA-CIA mice even though most others were downregulated.

### Potent NO burst occurred during development towards arthritis and NO was reversely correlated with SpO2

**Figure 4:**
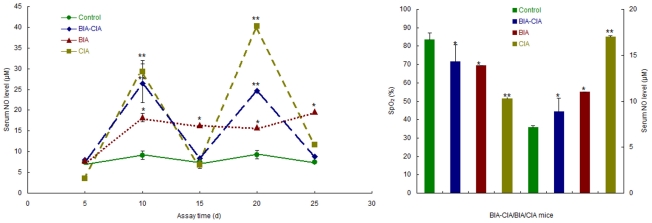
Dynamic monitoring of NO production and comparative analysis of relationship between NO and SpO_2_ in BIA, CIA and BIA-CIA mice. A. Time-course detection of serum NO levels in BIA, CIA and BIA-CIA mice (*n* = 3). Sampling and detection were conducted every 5 d and until 25 d during modeling; B. Measurement of SpO_2_ and NO in BIA, CIA and BIA-CIA mice (*n* = 4). Sampling and detection were conducted after modeling for 3 d in CIA and BIA-CIA mice or after modeling for 28 d in BIA mice. The singular asterisk (*) represents statistically significant difference from the control (*P*<0.05); and double asterisks (**) indicate statistically very significant difference from the control (*P*<0.01).

To monitor the dynamic change of NO production during live bacterial feeding and/or CII-CFA immunization, we determined the serum NO level, in a time-course manner, in modeling mice. Live bacterial feeding allowed the gradual elevation and subsequent maintenance of a steady-state NO level, whereas CII-CFA immunization led to the formation of double high NO peaks, occurring immediately after the primary challenging on the 1st d and boosting on the 21st d. Interestingly, BIA-CIA mice exhibited a similar pattern of peaked NO release with CIA mice although their NO levels were relatively lower than CIA mice (Figure 4A). Albeit in a distinct fashion and at a different extent, it was common that potent NO burst had been triggered among all modeling mice. As seen from the figure, much more NO was detected in CIA mice (40 µM) than in BIA-CIA mice (25 µM) or in BIA mice (20 µM) as compared in their highest levels. These results clarified that CII (alloantigen) and live bacteria or dead bacteria in CFA (xenoantigen) could stimulate an inducible release of NO, thereby exerting a pathogenic effect.

To confirm the assumption of NO burst leading to hypoxia, we monitored the fluctuation of SpO2 around the hind paws of modeling mice. A dramatic decrease of SpO2 was determined within erythematous and edematous paws of CIA mice. In contrast, only a slight decrease of SpO2 was measured in BIA-CIA and BIA mice (Figure 4B). Interestingly, lower SpO2 was correlated with higher NO and *vice versa* in those arthritic mice. For instance, 7 µM NO versus 80% SpO2 in control mice and 17 µM NO versus 50% SpO2 in CIA mice were detected, respectively. A similar relationship between NO and SpO2 also existed in mice with live bacterial feeding for 28 d, in which control mice had a lower NO level (7.14 µM) and a higher SpO2 value (83.5%), whereas mice had a higher NO level (11.03 µM) and a lower SpO2 value (69.5%).

### Infection or immunization led to NO-correlated elevation of LA and upregulation of HIF-1α and VEGF

**Figure 5:**
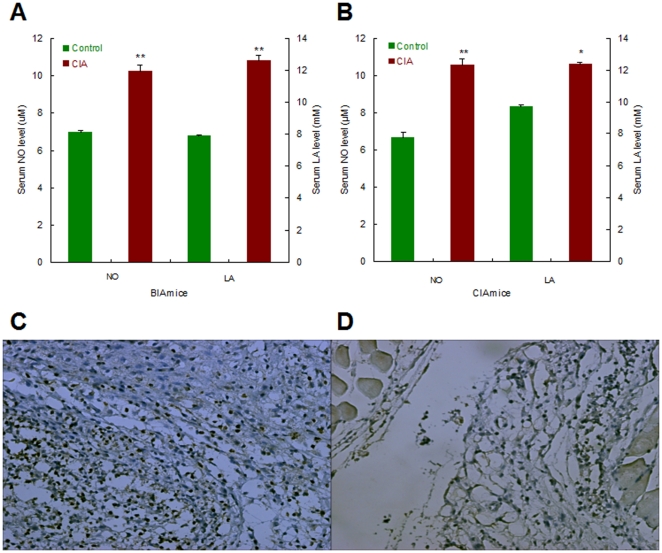
NO-driven hypoxia and angiogenesis in BIA and CIA mice. A. Serum NO and LA levels in BIA mice (*n* = 3); B. Serum NO and LA levels in CIA mice (*n* = 3); C. Immunohistochemical staining against HIF-1α in the articular synovium of CIA mice (×200); D. Immunohistochemical staining against VEGF in the articular synovium of CIA mice (×200). Sampling and detection were conducted after modeling for 3 d in CIA mice. The singular asterisk (*) represents statistically significant difference from the control (*P*<0.05); and double asterisks (**) indicate statistically very significant difference from the control (*P*<0.01).

To find out more direct evidence of NO-driven hypoxia, we detected the serum levels of NO and LA, an end glycolytic product, in BIA and CIA mice. A tight correlation of inducible NO with increased LA was observed in mice with daily bacterial feeding for 28 d or after 2 d of intra-articular CFA injection (Figure 5A and B). This result indicated that glycolysis for anaerobic degradation of carbohydrates must be enhanced following NO-driven hypoxia because it had eventually led to LA accumulation in blood.

Furthermore, we also investigated the overexpression profiles of angiogenesis-responsible genes in CIA mice. Consequently, both HIF-1α and VEGF were upregulated in CIA mice after intra-articular CFA injection (Figure 5C and D). Interestingly, immunohistochemical staining of articular sections was dependent on the amount of NO because subcutaneous injection of mice with sodium nitroprusside (SNP), an exogenous NO donor compound, enabled stronger staining for HIF-1α and VEGF in the hypoderm. The positive signals from HIF-1α and VEGF were remarkably enhanced in the hypoderm injected with 20 µg of SNP, up to seven folds and four folds, respectively (Table 3).

**Table 3:** SNP-induced overexpression of HIF-1α and VEGF in the SNP-injected hypoderm of mice (*n* = 3).

Treatment	Surface density(µm)	Positive unit (PU)	Immunohistochemical staining strength
HIF-1α			
Control	0.063±0.05	33.590±6.85	2.025±1.33
2 µg SNP (10 µg/ml)	0.358±0.06	18.660±3.60	6.694±1.82*
10 µg SNP (50 µg/ml)	0.242±0.06	31.003±3.69	7.493±2.16*
20 µg SNP (100 µg/ml)	0.410±0.04	34.410±2.94	14.067±1.23**
VEGF			
Control	0.087±0.01	24.867±1.66	2.169±0.39
2 µg SNP (10 µg/ml)	0.229±0.04	19.207±7.60	4.219±1.27*
10 µg SNP (50 µg/ml)	0.222±0.08	22.077±6.87	4.646±0.88*
20 µg SNP (100 µg/ml)	0.310±0.08	26.090±0.87	8.105±2.05**

Note: The immunohistochemical analysis was performed after 12 d for hypodermal sections of mice with daily injection of SNP (once a day), and the injected valume of SNP was 200 µl in all groups of treatments. The immunohistochemical staining strength was calculated from the formula of the surface density×the positive unit (PU), where the surface density represents the total area of positive loci/the total area for calculation. The singular asterisk (*) represents statistically significant difference from the control (*P*<0.05); and double asterisks (**) indicate statistically very significant difference from the control (*P*<0.01).

From above results, it was clearly informed that either endogenous NO provoked by immune activation or exogenous NO supplied by SNP injection could equally induce articular angiogenesis via driving synovial hypoxia that was characterized by lower SpO_2_ values and higher LA levels in blood.

### Administration of SNP mimicked inflammatory arthritis seen in BIA or CIA mice

**Figure 6:**
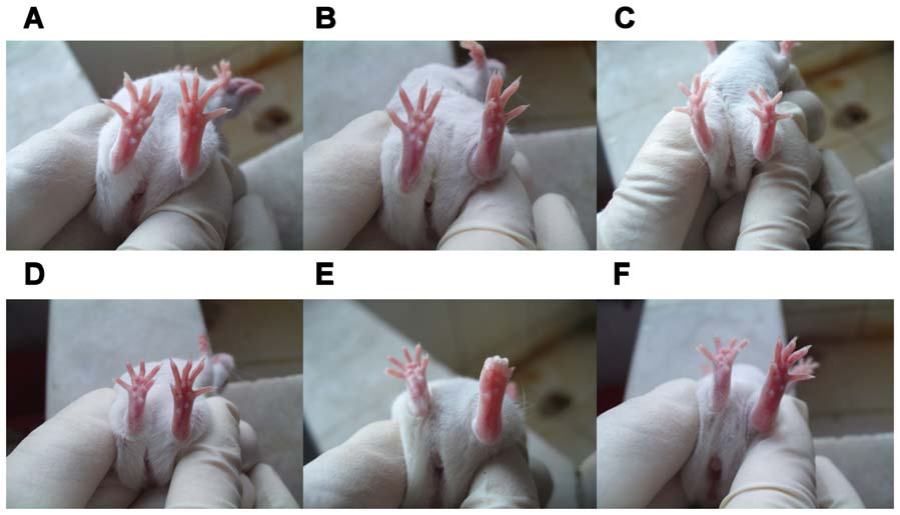
Articular inflammatory manifestations in mice after intra-articular injection with SNP, CII-CFA, or combined SNP and CII-CFA. A. Control; B. 20µ g SNP-injected mice; C. 40 µg CII-CFA-injected mice; D. 20 µg SNP+40 µg CII-CFA-injected mice; E. 4 µg CII-CFA-injected mice; F. 20 µg SNP+4 µg CII-CFA-injected mice. All photographes were taken after injection with SNP, CII-CFA, or combined SNP and CII-CFA for 1 d.

To address the implication of NO as an initiating factor of inflammatory arthritis, we simply administered mice with SNP by intra-articular injection. Fascinatingly, a single injection with SNP could give rise to a distinguishable manifestation of edema on the injected paw after only 1 d, legitimating a causitive effector leading to inflammatory articular lesions (Figure 6A and B). As expectation, similar inflammatory arthritis was observed in mice injected with different amounts of CII-CFA (Figure 6C and D). However, co-injection of mice with SNP and CII-CFA had induced more severe arthritis (Figure 6E and F). These results straightly demonstrated that exogenous NO could effectively serve as an inducer of synovitis, and such a procedure of SNP-induced acute arthritis might represent a novel and rapid approach for modeling of RA-like arthritis in mice.

From measurement of the critical hypoxia parameter SpO2, it was noticed that very low values of SpO2 (55–57%) were induced in mice after injection with SNP regardless treatment with or without CII-CFA. At the same time, different amounts of CII-CFA could result in moderately lower values of SpO2 (62–66%), which were lower than control mice (82%) but higher than SNP-injected mice (Table 4). We were also aware from the table that the extent of hypoxia seemed not tightly correlated with the severity of articular inflammation. For example, a higher SpO2 value (62%) rendered more significant edema on the paw injected with CII-CFA. This result might imply that CII-CFA-triggered immune activation must be implicated for the progression of articular inflammation.

**Table 4:** Comparison of SpO_2_ in articulates of mice after intra-articular injections by SNP, CII-CFA, or SNP+CII-CFA (*n* = 3).

Treatment	SpO_2_ (%)
Control	81.67±1.15
20 µg SNP (100 µg/ml)	56.67±2.08**
40 µg CII-CFA (200 µg/ml)	61.67±3.79*
40 µg CII-CFA+20 µg SNP (200 µg/ml CII-CFA+100 µg/ml SNP)	54.67±2.89**
4 µg CII-CFA (20 µg/ml)	64.33±2.52*
4 µg CII-CFA+20 µg SNP (20 µg/ml CII-CFA+100 µg/ml SNP)	54.67±0.58**

Note: SpO_2_ was measured after 3 d for articulates of mice with intra-articular SNP, CII-CFA, or SNP+CII-CFA. The injected valume of each drug or a drug combination was 200 µl in all groups of treatments. The singular asterisk (*) represents statistically significant difference from the control (*P*<0.05); and double asterisks (**) indicate statistically very significant difference from the control (*P*<0.01).

### Anti-bacteria and NOS inhibition abrogated infection- or immunization- triggered NO production and mitigated hypoxic consequences

**Figure 7:**
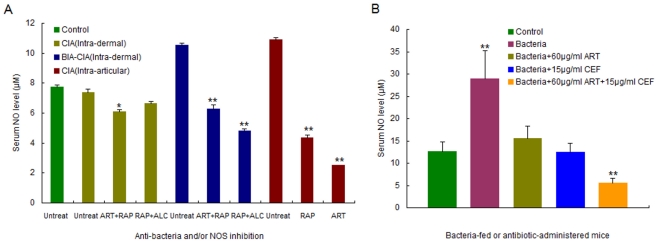
Abrogation of NO production after anti-bacteria and/or NOS inhibition in BIA, CIA and BIA-CIA mice. A. Serum NO levels in drug-administered BIA, CIA and BIA-CIA mice (*n* = 3). The serum NO level was determined after anti-bacteria and/or NOS inhibition of modeling mice by 60 µg/ml artesunate, 50 µg/ml rapamycin, 15% alcohol or a combination of drugs; B. Serum NO levels in bacteria-fed or antibiotic-administered mice (*n* = 10). The serum NO level was determined after live bacterial feeding for 7 d and injecting artesunate, cefotaxime, or the combination of artesunate with cefotaxime for 3 d (twice a day). ART: artesunate; CEF: cefotaxime; RAP: rapamycin; ALC: alcohol. The singular asterisk (*) represents statistically significant difference from the control (*P*<0.05); and double asterisks (**) indicate statistically very significant difference from the control (*P*<0.01).

To validate bacterial infection is an inducer of NO generation, we determined the serum level of NO in mice after live bacterial feeding for 7 d as well as after subcutaneous injection of the NOS inhibitor artesunate, the antibiotic cefotaxime, or the combination of artesunate with cefotaxime for 3 d (twice a day). As illustrated in Figure 7A, artesunate, cefotaxime, or artesunate+cefotaxime considerably decreased NO production, and the serum level of NO in the combined treatment group was even lower than the control. Conceivably, artesunate decreased the serum NO levels due to the inhibition of NOS activity even though infection still existed. Cefotaxime allowed a serum level of NO equal to the control because of infection blocking. The combined treatment of bacteria-fed mice by artesunate with cefotaxime lowered the serum NO level below that seen in the control owing to dual effects of infection suppression and NOS inhibition. These results verified that persistent gastrointestinal infection did trigger potent NO burst in mice, but this outcome could be mitigated or abrogated by anti-bacteria and/or NOS inhibition.

Because no significant inflammatory symptoms occurred after a short period (7 d) of bacterial feeding in mice, the severity of bacteria-induced synovitis could not be scored and a potential anti-arthritic effect of artesunate and/or cefotaxime was also difficult to be evaluated. However, therapeutic efficacy of cefotaxime, phytol, alcohol, or a combination on BIA could be reflected from either the amelioration of morphological alterations in mice with live bacterial feeding for more than one month or the normalization of hypoxic parameters including NO, LA and SpO_2_ in mice at any time points during live bacterial feeding. Table 5 listed those data of hypoxic parameters determined before and after anti-bacterial or other treatments in BIA mice. Interestingly, anti-bacterial treatment by cefotaxime injection or diluted alcohol drinking demonstrated an eradication of hypoxic consequences, whereas the oxidative burst inducer phytol showed only a partial normalization of hypoxic parameters.

**Table 5:** Measurement of hypoxic parameters for evaluation of cefotaxime on bacteria fed mice (*n* = 4).

Treatment	NO (µM)	LA (mM)	SpO_2_ (%)
Control	7.15±0.30	7.88±0.13	83.00±1.83
BIA (one-month live bacterial feeding)	9.87±0.48*	12.58±0.19**	70.50±2.08*
BIA treated by 3 µg CEF (15 µg/ml) for 3 d	2.97±0.13**	7.83±0.07	80.50±0.71
BIA treated by 3 µg CEF (15 µg/ml) for 5 d	3.12±0.15**	7.91±0.05	82.50±0.71
BIA treated by 12 µg PTL (60 µg/ml) for 3 d	5.14±0.13*	6.79±0.05	69.50±0.71*
BIA treated by 12 µg PTL (60 µg/ml) for 5 d	4.62±0.15*	11.50±0.05**	78.50±0.71
BIA treated by 3 µg CEF (15 µg/ml)+12 µg PTL (60 µg/ml) for 3 d	7.57±0.26	14.36±0.05**	75.50±0.71
BIA treated by 3 µg CEF (15 µg/ml)+12 µg PTL (60 µg/ml) for 5 d	4.84±0.15*	13.57±0.05**	80.50±0.71
BIA treated by 15% ACL+12 µg PTL (60 µg/ml) for 3 d	7.30±0.13	6.79±0.05	76.50±0.71
BIA treated by 15% ACL+12 µg PTL (60 µg/ml) for 5 d	5.91±0.15*	8.32±0.05	76.50±0.71

Note: NO, LA, and SpO_2_ were measured in mice with live bacterial feeding or after drug administration for 3 d or 5 d. The injected valume of each drug or a drug combination was 200 µl in all groups of treatments. The singular asterisk (*) represents statistically significant difference from the control (*P*<0.05); and double asterisks (**) indicate statistically very significant difference from the control (*P*<0.01). CEF: cefotaxime for subcutaneous injection; PTL: phytol for intra-articular injection; ACL: alcohol for drinking.

In CIA mice established by intra-dermal or intra-articular CII-CFA immunization, NO was dramatically decreased to a level lower than the control after artesunate or rapamycin administration. Combined application of artesunate with rapamycin, or rapamycin with alcohol in BIA-CIA mice led to considerably repressed NO production. In particular, an extremely lower NO level (0.448 µM) was detected in one artesunate-administered CIA mouse. In contrast, untreated BIA-CIA and CIA mice exhibited much higher NO levels, up to 10 µM in the maximum (Figure 7B). Intriguely, untreated CIA mice even gave rise to a low NO level almost equal to that of the control, which might be attributed to a decay effect of CII-CFA-triggered NO production after longer duration of post-immunization. Actually, three weeks had passed from CII-CFA boosting to NO detection.

### NO scavenging also mitigated articular inflammation albeit to a different extent

**Figure 8:**
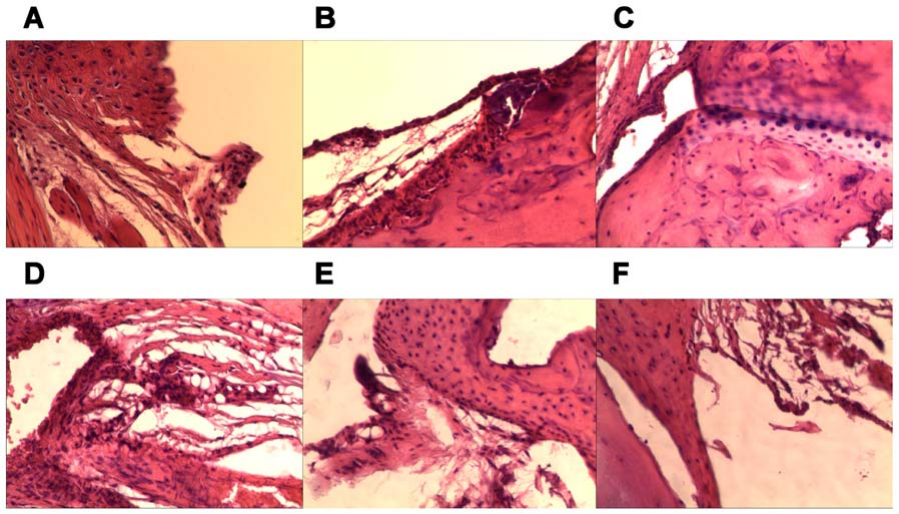
Histological amelioration of synovitis by anti-arthritis therapies in CIA and BIA-CIA mice (HE staining, ×200). A and B. Pre-treatment of CIA (intra-articular CII-CFA injection) mice with 60 µg/ml artesunate or 50 µg/ml rapamycin; C and D. Post-treatment of CIA (intra-dermal CII-CFA injection) mice with 60 µg/ml artesunate and 50 µg/ml rapamycin, or 50 µg/ml rapamycin and 15% alcohol; E and F. Post-treatment of BIA-CIA (intra-dermal CII-CFA injection) mice with 60 µg/ml artesunate and 50 µg/ml rapamycin, or 50 µg/ml rapamycin and 15% alcohol. HE staining of sections and microscopic analysis were carried out by sampling hind paws of mice after therapy completion.

According to morphological and histochemical identifications of the inflamed synovium, amelioration of articular synovitis could be practically confirmed in anti-arthritic drug-treated mice although their merits were not on average levels. In mice with intra-articular CII-CFA injection, synchronous administration of artesunate or rapamycin had kept the synovium intact from intimal hyperplasia and subintimal fibrosis. However, dispersed synovial infiltration by inflammatory lymphocytes was still observed in the artesunate-treated group, but not in the rapamycin-treated group (Figure 8A and B). These results indicated that artesunate as an inhibitor of NOS only suppressed hyperplasia, while rapamycin as an immunosuppressant that can block immune activation-dependent NO production could attenuate both hyperplasia and infiltration.

Among CIA mice, post-treatment by artesunate and rapamycin aborted both intimal hyperplasia and subintimal fibrosis, but failed to recover the synovium from mild and dispersed inflammatory infiltration by mononucleates. On the other hand, co-administration of CIA mice with rapamycin and alcohol did not hamper the progression to local synovial hyperplasia and mild lymphocytic infiltration (Figure 8C and D). Post-injection of BIA-CIA mice with artesunate and rapamycin abolished subintimal fibrosis and inflammatory infiltration, but intimal hyperplasia could not be prohibited completely. In contrast, rapamycin and alcohol treatment could remit the hyperplasic synovium, but did not inhibit progression to mild and dispersed inflammatory infiltration (Figure 8E and F).

Above outcomes of therapeutic regimens demonstrated, therefore, that pre-treatment prior to NO generation was more effective than post-treatment after NO generation. In other words, synovial damage made prior to drug administration failed to be ameliorated by any regimen of above post-treatments. These results conclusively indicated that NO must represent an initiator of RA-like arthritis, mainly responsible for synovial hyperplasia, so NO-initiated synovial lesions might not be reversible by any NOS inhibitors.

## Discussion

As a gaseous free radical with pleiotropic functions in biological systems, NO is produced from the oxidation of L-arginine to L-citrulline by the catalysis of either isoform of three types of NOS: inducible NOS (iNOS), endothelial NOS (eNOS) and neuronal NOS (nNOS) [25]. Through generating the inducible NO during pathogenic infection, iNOS is involved in immune attack against active invaders. For instance, bacterial infection of human colon epithelial cells rapidly upregulates iNOS expression and enhances NO production [26]. Apart from gastrointestinal infection following live bacterial feeding, however, we found that immunization of mice with CII-CFA could also provoke potent NO burst, hence suggesting that NO production was dependent on immune activation regardless of pathogenic infection.

Arthritis modeling in mice has been generally considered to require heat-killed *Mycobacterium tuberculosis*-containing CFA in addition to CII, strongly implying that anti-CII responses are insufficient to induce arthritis in mice. Some authors thought that anti-CII reactivity might be a consequence of inflammation rather than the cause [27]. This is why so many kinds of autoantibodies were formed in RA patients or experimental arthritic rodents, such as those against citrullinated proteins, glucose-6-phosphate isomerase, integrin, and fibrin, etc. [28]–[31]. So we assume that CII is probably indispensable for modeling RA, at least inducing synovitis, in mice. Indeed, we have successfully induced acute arthritis in mice by intra-articular injection with CFA alone instead of CII-CFA (unpublished results). Therefore, we draw a conclusion here that any immune activators including autoantigens and bacteria, either live or dead, could evoke the corresponding immune responses and confer inflammatory lesions by triggering NO generation and driving hypoxic consequences.

The mucosal response to enteric infection includes the production of chemoattractant cytokines (chemokines), anti-inflammatory cytokines, and pro-inflammatory cytokines, in which TNFα is the most important pro-inflammatory cytokine that can amplify the epithelial immune response to bacterial infection [32]. TNFα, IL-1β and other pro-inflammatory cytokines can upregulate NOS in chondrocytes and synovial cells of osteoarthritis [33].

We have also observed the global activation of more than 30 kinds of chemokines, cytokines and receptors in BIA and CIA mice, including TNFα, IL-1β, IL-6, M-CSF, and MCP-1, which were capable of driving a complex and chronic inflammatory process [34]. In the present study, an experimental outcome that TNFα was downregulated in CIA mice was puzzling, but it was most likely attributed to the attenuation of immune responses to CII-CFA after experenced for several weeks of immunization. As noticed, NO levels were declined twice (15^th^ d and 25^th^ d) in CIA mice to levels approaching to those seen in the control during modeling. In fact, sampling for cytokine chip profiling was carried out after modeling for 30 d. Indeed, a higher serum level of TNFα, as two folds as the control, was measured in mice after 3 d of intra-articular injection with CII-CFA (unpublished results).

Although the implication of NO in the pathogenesis of experimental arthritis is poorly understood, CII-CFA-triggered NO burst had been noticed in CIA mice [35]. We also observed that two NO peaks were formed immediately after primary challenging and boosting with CII-CFA, whereas a stable level of NO higher than the control maintained in the course of daily live bacterial feeding. Albeit in a distinct manner, either CII-CFA or bacteria did provoke NO production, permanently or transiently, in BIA and CIA mice. Nevertheless, whether an extraordinarily higher level of NO only initiating or even controlling the progression of inflammatory processes is an open question. On the other hand, we also found that co-treatment by CII-CFA immunization with live bacterial feeding could allow the global downregulation of pro-inflammatory cytokines including TNFα, IL-1β and IL-6, hence suggesting an immunosuppressive role of bacteria upon CII-CFA-activated immune responses. In consistence with our findings, *Schistosoma japonicum* infection has been reported to significantly attenuate the clinical signs, to reduce the histological damage, to alter the humoral immune responses, and to inhibit the splenocyte proliferation in CIA mice [36]. Additionally, immunosuppression by bacteria has been addressed in a recently published review [37]. Therefore, it is believed that arthritic development might be mostly dependent on systemic immune activation, and immunosuppression can, of couse, alleviate inflammatory arthritis.

Rapamycin is a well-known immunosuppressant that reduces pannus formation, cartilage erosion and joint damage in rats with adjuvant-induced arthritis [38]. Artesunate also plays an anti-arthritic role in CIA mice [39]. Even though the pharmacological mechanisms of rapamycin and artesunate as therapeutic agents of arthritis are thought to be multifaceted, no experimental records have been documented regarding their suppression on NO production and potential preventive effects on arthritis. In the present study, we have linked, for the first time, the abrogation of inflammatory arthritis with the repression of NO generation in mice that had accepted rapamycin and/or artesunate prior to modeling. Synchronous administration of such anti-arthritic drugs to CII-CFA immunized mice could effectively block the onset of synovitis or hamper its aggravation towards arthritis. However, drug combinations for post-treatment in the present regimen never completely repaired the damaged synovium of modeling mice.

Nevertheless, anti-bacterial and/or pro-apoptotic treatments by cefotaxime, alcohol, phytol or a combination of multi-drugs in live bacterial feeding mice seemed to improve the inflamed synovium at a certain extent, being reflected from the normalization of hypoxic parameters, NO, LA, and SpO_2_. Cefotaxime could dramatically decrease the level of NO, LA, and SpO_2_ to control levels. Phytol, alcohol, or their combination could more or less normalize those hypoxic parameters. Support evidence to a conclusion of diluted alcohol being beneficial to RA patients has been reported in a clinical cohort that alcohol consumption is inversely associated with risk and severity of RA [40]. On the other hand, phytol as an oxidative burst inducer has been used for treatment of experimental arthritis, from which decreased autoimmune responses and amelioration of both the acute and chronic phases of arthritis have been reported in rats [41].

From the histochemical analysis of articular sections prepared from the synovial and articular tissue of BIA and CIA mice, we observed remarkable synovial hyperplasia, an indicative hint of angiogenesis and tumorigenesis. It has been previously filed that angiogenesis is an early event in the inflammatory joint that is important in enabling activated monocytes to enter the synovium and expand it into a pannus via endothelial cells by active recruitment, resulting in cartilage degradation and bone destruction [42]. Hypoxia is capable of inducing the expression of angiogenesis-related genes including hypoxia inducible factor 1 alpha (HIF-1α) and vascular endothelial growth factor (VEGF) [43]. Alternatively, NO can also activate HIF-1α under normoxic conditions [44], hence implying NO as a hypoxic inducer. We found that supplementation with exogenous NO through SNP injection could effectively induce synovitis, and also cause hypodermal neoplasia in mice (unpublished results), thereby suggesting that NO-driven angiogenesis is a setpoint towards hyperplasia.

The synovium itself covering the articular organ is a relatively hypoxic tissue, in which oxygen tension in cartilage ranges from 7% (53 mm Hg) in the superficial layer to less than 1% (7.6 mm Hg) in the deep zone [45]. As a result, hemoglobin carrying with NO can not supply sufficient O2 to the synovium under hypoxic conditions, and NO can also accelerate its own consumption by increasing its entry into red blood cells [46]. NO inhibits the mitochondrial enzyme cytochrome c oxidase (complex IV) in competition with O2, leading to so-called “metabolic hypoxia” – a situation in which, although oxygen is available, the cell is unable to utilize it [47]. High levels of NO inhibit cell respiration by binding to cytochrome c oxidase, whereas slow and small-scaled NO release can stimulate mitochondrial biogenesis in diverse cell types [48]. Our results indicated that enhanced NO production was correlated with lower SpO2 values, seeming to reveal a direct consequence of NO leading to SpO2. It is most likely that NO drives hypoxia by competing O2 that has been bound to the hemoglobin or preferentially occupying the O_2_-binding site of the non-oxygenated hemoglobin. Such knowledge should convince us that NO conveys angiogenesis and hyperplasia via creating a hypoxic microenvironment.

Due to NO-mediated hypoxic effects, blood carbohydrates were anaerobically catabolized and glycolytic metabolites were necessarily converted to LA, which would be accumulated in the systemic blood stream unless oxygen supply was rehabilitated. In the present investigation, we monitored the dynamic changes of serum NO and LA levels synchronously, and found a proportional fluctuation of NO with LA in arthritic modeling mice. The serum level of LA is obviously a new reference parameter for quantifying hypoxia in addition to SpO2, and LA can also indicate the transversion from hypoxia to normoxia. Furthermore, it has been known that hypoxia can activate the responded transcriptional factor HIF-1α, which in turn binds to the promoter of VEGF gene for starting transcription and translation [49]. We detected the overexpression of HIF-1α and VEGF in the inflamed synovium of acute CIA mice albeit only in a moderate degree. When exogenous NO derived from SNP was supplemented into the hypoderm of mice, much higher expression levels of HIF-1α and VEGF than those seen in acute CIA mice were detected, thereby suggesting a reliable relevance of NO-driven overexpression of HIF-1α and VEGF with synovial as well as systemic angiogenesis during tissue hyperplasic induction.

In view of different roles playing by NO and pro-inflammatory cytokines, we believe that both of which are important in determining the initiation and progression of inflammatory arthritis in mice. It is possibly that NO is mainly responsible for synovial hyperplasia, whereas pro-inflammatory cytokines are relevant to inflammatory infiltration. NO may induce synovial angiogenesis and hyperplasia by NO-mediated hypoxia, which can subsequently guide pro-inflammatory cytokines penetrating deeply into the synovium along with the newborn blood vessel [50]. Our results in experimental arthritis in mice indicated that NO promoted angiogenesis by activating HIF-1α and VEGF and in turn mitigated glycolysis after enhanced angiogenesis. These results would become a basis for treatment of arthritis by the inhibition of NO-driven angiogenesis. Currently published data have demonstrated that anti-VEGF treatment by bevacizumab reduces blood supply, increases glycolytic metabolites, and promotes tumor metastasis in glioblastoma [51], underlining that no amelioration would be reached if inflammation-originated hypoxia has not been alleviated or abrogated.

In conclusion, our present study have answered a long-term unanswered question about the association of distal or systemic infection with inflammatory arthritis: gastrointestinal infection can serve as an etiological initiator of inflammatory arthritis by dually upregulating pro-inflammatory cytokines to allow lymphocytic infiltration and triggering NO to drive synovial hypoxia and hyperplasia. These achievements should shed light on the prophylactic and therapeutic interventions of RA and other human autoimmune diseases in the future clinical trials.

## Materials and Methods

### Induction of experimental arthritis in mice

Male KM mice (7–8 weeks old, 20–22 g) were provided by The Experimental Animal Center of Guangzhou University of Chinese Medicine, China. All animal experiments in the present study were carried out in strict accordance with the recommendations in the Guide for the Care and Use of Laboratory Animals of the National Institutes of Health (NIH), USA. The protocol was approved by the Animal Care Welfare Committee of Guangzhou University of Chinese Medicine (Permit Number: SPF-2011007). To perform gastrointestinal bacterial infection, an overnight culture of the non-pathogenic *E. coli* strain DH5α in 10^8^ cells was mixed with food for daily feeding of mice. To conduct intra-articular or intra-dermal CII-CFA immunization, CII (Sigma-Aldrich, USA) was dissolved in 0.1 M acetic acid (2 mg/ml) and emulsified with equal amounts of CFA (Sigma-Aldrich, USA) for injection into the unilateral ankle articular cavity or at the tail base, and primarily challenged mice were boosted with the same CII-FCA mixture after three weeks [52]. The severity of inflammatory arthritis was measured by scoring each limb from 0 to 4 grades and by summing up the clinical scores of four limbs: 0 = normal; 1 = erythema or edema of one or several digits; 2 = erythema and moderate edema extending from the ankle to the mid-foot (tarsals); 3 = erythema and severe edema extending from the ankle to the metatarsal joints; and 4 = complete erythema and edema encompassing the ankle, foot and digits, resulting in deformity and/or ankylosis [53].

### Determination of serum NO, LA and blood SpO2

NO and LA were determined using commercially available kits (Nanjing Jiancheng, China) and according to the manufacturer's instruction. The serum level of NO was calculated from the formula: NO (µM) = (OD550 test-OD550 blank/OD550 standard-OD550 blank)×C standard (20 µM)×dilution fold (1 fold). The serum level of LA was calculated from the formula: LA (mM) = (OD530 test-OD530 blank/OD530 standard-OD530 blank)×C standard (3 mM)×dilution fold (7 folds). Blood SpO2 was measured by inserting a hind paw or the injected paw into the cavity of a blood SpO2 photometer MD300C (Beijing Chaosi Electronics, China). The value showing on the LED screen is SpO2 (%).

### Protein extraction and cytokine antibody chip analysis

Protein extraction from blood cells by Cell & Tissue Protein Extraction Reagent (KangChen KC-415) was conducted according to the manufacturer's instruction. Cytokine antibody array was carried out by KangChen Bio-Tech, Shanghai, China using RayBio® Mouse Cytokine Antibody Array and according to the manufacturer's manual as following steps: Blocking and incubation, detection, and result interpretation (http://www.kangchen.com.cn/support/Supportmain.asp?ID=129).

### Articular sampling and histochemical staining

Formalin-fixed and paraffin-embedded joint tissues (including synovium and cartilage) were cut at a thickness of 5 µm. Sections were deparaffinized by xylene, re-hydrated by gradient alcohol, and washed in distilled water. After hematoxylin staining, washing in running tap water, differentiation in 1% acid alcohol, washing in running tap water, bluing in 1% ammonia, washing in running tap water, and rinsing in 95% alcohol was performed. After eosin counter staining, dehydration through gradient alcohol, clearance in xylene, and mount with xylene-based mounting medium. Semi-quantitative analysis of sections was carried out by light microscopy equipped with a camera using a modified non-parametric scoring system [54], in which 0, +, ++, +++, and++++for each criteria of normal, intimal hyperplasia, subintimal fibrosis, lymphocytic infiltration, and articular damage were evaluated, respectively.

### Immunohistochemical quantification

Formalin-fixation, paraffin-embedment and deparaffinization were the same as HE staining procedure described above. Sections were incubated at room temperature with 3% H_2_O_2_ to block endogenous peroxidases, and then repaired in boiling citric acid. After washing in phosphate-buffered solution (PBS), sections were blocked by 2% bovine serum albumin (BSA) and incubated with 1∶100 diluted primary antibodies at 37°C for 1 h. After washing with PBS, sections were incubated with biotinylated secondary antibodies at 37°C for 20 min. After washing with PBS, sections were incubated with diaminobenzidine (DAB) for 1–5 min. After rinsing with tap water, sections were counter stained by hematoxylin. After completion of dehydration, clearance and mounting, pictures were taken under the microscope (OLYMUPUS BX-51) and immunoquantitatively analyzed by the MIAS microimage analysis system (Beijing, China). The quantitative result was calculated based on the formula: The immunohistochemical staining strength = the surface density×the positive unit (PU), where the surface density represents the total area of positive loci/the total area for calculation.

### Statistical analysis

The difference of value in each assessment was analyzed by Student *t* test. A *P* value of less than 0.05 is statistically significant (*), and a *P* value of less than 0.01 is statistically very significant (**). All data were analyzed using SPSS version 10.0 for Windows.
